# 4231 Identifying Educational Needs of Researchers and Health System and Health Agency Leaders in the Science of Implementation and Improvement: Report from California CTSAs

**DOI:** 10.1017/cts.2020.397

**Published:** 2020-07-29

**Authors:** Moira Inkelas, Brian Mittman, Margaret Handley, Miriam Bender, Brad Pollock, Oanh Nguyen, Greg Aarons, Michael Cousineau, Rachael Sak

**Affiliations:** 1 David Geffen School of Medicine at UCLA; 2 Kaiser Permanente Southern California; 3 University of California, San Francisco; 4 University of California, Irvine; 5 University of California, Davis; 6 University of California, San Diego; 7 University of Southern California; 8 University of California Office of the President

## Abstract

OBJECTIVES/GOALS: We conducted interviews with investigators, clinicians, and health system and health agency leaders to assess regional educational needs in implementation and improvement science, including content (knowledge and skill), format, experiential learning, and mentoring, to identify barriers and guide planning. METHODS/STUDY POPULATION: Five CTSAs in the University of California Biomedical Research Acceleration, Integration, & Development consortium (UC BRAID) plus a fifth affiliated CTSA developed a common protocol and interviewed 31 California-based learners (current fellows, early and mid-career investigators, clinicians, and health agency personnel) and system leaders from health care and health agencies. Interviews focused on impact goals, educational needs in dissemination, implementation, and improvement (DII) science, challenges in DII research, preferred learning formats, desired proficiencies and skills, and barriers such as cost, time, awareness, terminology, and suitability and availability of training. A rapid review of literature identified potential domains of knowledge and skills for a proposed curriculum. RESULTS/ANTICIPATED RESULTS: Areas of emphasis varied among interviewees; identified learning needs differed between traditional research perspectives (emphasizing areas such as partner engagement, grant writing, frameworks, study design) and applied perspectives (emphasizing areas such as managing change, complex systems, learning system capacity). Learners had a range of preferences; most interviewees desired formats that are longitudinal, experiential, applied, cooperative, and affordable. Variation in knowledge of, and interpretations of, DII terms and goals limited the ability of some interviewees to specify educational needs. A synthesis reveals areas for potential future co-development and networked approaches to regional training and capacity enhancement. DISCUSSION/SIGNIFICANCE OF IMPACT: In response to a rapidly changing health landscape, our academic health systems are developing capabilities to improve care for their populations. Our work informs the training and education needs that are critical to translation at a system-wide level. Regional convenings can raise awareness while translational programs fill educational gaps.

